# Horse-Specific *Cryptosporidium* Genotype in Human with Crohn's Disease and Arthritis

**DOI:** 10.3201/eid2806.220064

**Published:** 2022-06

**Authors:** Żaneta Zajączkowska, Anna Baštýřová Brutovská, Katarzyna Akutko, John McEvoy, Bohumil Sak, Andrzej B. Hendrich, Błażej Łukianowski, Martin Kváč, Marta Kicia

**Affiliations:** Wroclaw Medical University, Wroclaw, Poland (Ż. Zajączkowska, K. Akutko, A.B. Hendrich, B. Łukianowski, M. Kicia);; Czech Academy of Sciences, České Budějovice, Czech Republic (A. Baštýřová Brutovská, B. Sak, M. Kváč);; North Dakota State University, Fargo, North Dakota, USA (J. McEvoy);; University of South Bohemia, České Budějovice (M. Kváč)

**Keywords:** cryptosporidiosis, transmission, Cryptosporidium, protist, parasite, genotype, horse-specific, horse, human, intestinal infections, enteric infections, Crohn’s disease, juvenile rheumatoid arthritis, zoonoses

## Abstract

We identified an unusual subtype of a *Cryptosporidium* sp. horse genotype as the cause of cryptosporidiosis in a 13-year-old girl in Poland who was undergoing immunosuppressive treatment for juvenile rheumatoid arthritis and Crohn’s disease. The same subtype was identified in a horse the girl had ridden.

*Cryptosporidium* spp. causes diarrheal disease that can become chronic and life-threatening in persons who have an immature or malfunctioning immune system. Humans are primarily infected with *C. hominis* and *C. parvum* (*1*), but can also be infected with other species and genotypes, including *Cryptosporidium* sp. horse genotype, which primarily infects horses and donkeys.

Molecular studies targeting the polymorphic 60-kD glycoprotein (*gp60*) gene have shown that humans and horses/donkeys are infected with different subtypes of the *Cryptosporidium* sp. horse genotype (*2*–*4*). We identified an unusual subtype of the *Cryptosporidium* sp. horse genotype as the cause of cryptosporidiosis in a 13-year-old girl receiving immunosuppressive treatment for juvenile rheumatoid arthritis and Crohn’s disease.

The Human Research Ethics Committee of Wroclaw Medical University approved use of diagnostic samples and corresponding patient data for this study (permit No. KB-24/2014). Written consent was provided by the parents of the child involved in the study.

The girl was being treated at the Wroclaw Medical University (Wroclaw, Poland) since 2009. On the basis of recommended criteria, she was given a diagnoses of rheumatoid arthritis when she was 3 years of age and Crohn’s disease when she was 5 years of age (*5*,*6*). She had received the immunosuppressant adalimumab since her rheumatoid arthritis diagnosis; that treatment continued after diagnosis of Crohn’s disease because of frequent relapses and lesions in the large bowel. In 2018, she was hospitalized because of recurrent gastrointestinal relapses, including diarrhea, fever (temperature >38.5°C), and abdominal cramps. Initial therapy included entocort (9 mg/d) and mesalizine (2,000 mg/d).

We tested stool samples for a panel of gastrointestinal bacteria (*Salmonella*, *Shigella*, *Campylobacter*, *Yersinia*, and *Clostridium difficile*). We also tested for parasitic protists, including microsporidia (*Encephalitozoon* spp., *Enterocytozoon bieneusi*), *Cryptosporidium* spp., *Giardia intestinalis*, and *Cyclospora cayetanensis*. Examination of stool showed *Cryptosporidium* oocysts (20,000‒60,000 oocysts/gram of stool (7). Test results for other gastrointestinal pathogens (bacteria and other parasitic protists) were negative. 

We used the small ribosomal subunit rRNA and *gp60* genes (*8*), respectively, to identify *Cryptosporidium* to the species/genotype and subgenotype levels. We purified amplicons (QIAquickR; QIAGEN, https://www.qiagen.com) and directly sequenced them in both directions (SeqMe, https://www.seqme.eu). We repeated amplification and sequencing of each locus 3 times. We aligned nucleotide sequences by using references from GenBank and MAFFT version 7 (https://mafft.cbrc.jp/alignment/software) and performed phylogenetic analysis by using the maximum-likelihood method in MEGAX (https://www.megasoftware.net). Phylogenetic analyses of small ribosomal subunit rRNA and *gp60* showed a *Cryptosporidium* sp. horse genotype belonging to the subtype family VIaA15G4 (Figure). 

**Figure:**
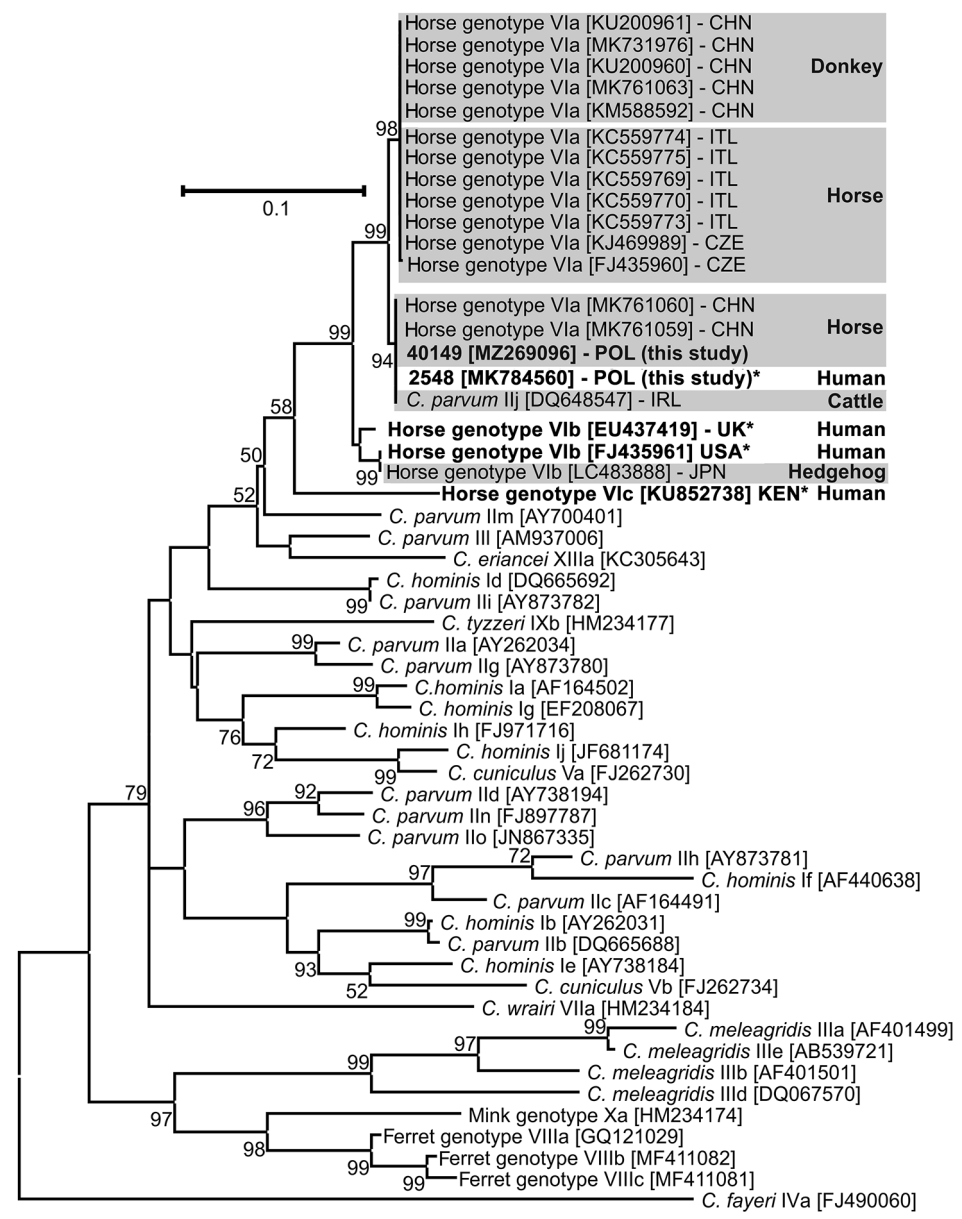
Maximum-likelihood tree based on partial sequences of 60-kD glycoprotein of *Cryptosporidium* spp. from a 13-year-old immunocompromised girl and a horse she rode in Poland (and reference sequences. Bold and asterisks indicate isolates reported from humans; gray shading indicates isolates reported from animals. The general time reversible model was applied using a discrete gamma distribution. Robustness of the phylogeny was tested with 1,000 bootstraps. Values along the branches indicate bootstrap values with >50% support. GenBank accession number are indicated in brackets. Country of origin of *Cryptosporidium* sp. horse genotype isolates is indicated by 3-letter International Organization for Standardization country abbreviation. Scale bar indicates nucleotide substitutions per site.

A follow-up medical interview showed that the patient rode a horse once per week at a riding stable; therefore, we collected fecal samples from all 10 horses at the stable and analyzed them for *Cryptosporidium* spp. None of the examined horses shed microscopically detectable *Cryptosporidium* oocysts or showed signs of cryptosporidiosis.

However, a horse ridden by the patient was PCR positive for a *Cryptosporidium* sp. horse genotype that had 100% identity with the isolate from the patient (GenBank accession nos. MK779952, MK784560, MZ255144, and MZ269096). The patient stopped attending the stable after the diagnosis, and symptoms resolved within 2 weeks without specific *Cryptosporidium* treatment. *Cryptosporidium* was not detectable in the patient 2 months after the diagnosis, and no exacerbations of underlying disease were observed during the 1-year follow-up period.

Documentation of direct animal-to-human transmission of *Cryptosporidium* spp. is rare. In most cases of infection by animal-specific *Cryptosporidium*, patients did not report direct contact with the host suspected of being the source of infection. Our findings demonstrate horse-to-human transmission of the *Cryptosporidium* sp. horse genotype VIa family, a subtype previously believed to be specific to horses and donkeys.

Crohn’s disease pathophysiology is closely linked to perturbations of the gut microbiome, but this disease and its causes remain poorly understood. Because of unexplained etiology, there is no specific treatment; immunosuppressive drugs are used to reduce inflammation, achieve remission, or prevent exacerbation. Immunosuppressive therapies cause increased susceptibility to opportunistic pathogens, such as *Cryptosporidium* spp., and limited data suggest that *Cryptosporidium* infection in inflammatory bowel disease (IBD) patients is not rare (*9*), despite protists being frequently overlooked in diagnostic testing. Manifestation of intestinal cryptosporidiosis might be confused with symptoms of Crohn’s disease and other IBD relapses.

The condition of the immune system is a critical determinant of the clinical course of *Cryptosporidium* infection. Despite immunosuppressive therapy, we observed complete clearance of the *Cryptosporidium* infection without specific treatment. In this regard, the infection was more similar to a case of self-limiting diarrhea in an immunocompetent person than to a chronic and life-threatening disease that is frequently associated with immunocompromised persons (*10*). Our findings suggest that the combination of Crohn’s disease and rheumatoid arthritis and immunomodulatory treatment might increase likelihood of infection with *Cryptosporidium* spp. that are not commonly infectious for humans.

In summary, we show the need for considering atypical sources of *Cryptosporidium* infection in persons with IBD who are undergoing immunosuppressive therapy. A necessary first step is to expand diagnostic testing of IBD patients to include opportunistic protists, such as *Cryptosporidium* spp. Once diagnosis is confirmed, genotyping of isolates can be used to help identify the source of infection, which is critical to preventing disease recurrence.
